# Prostate Cancer Phenotype Influences Bone Mineralization at Metastasis: A Study Using an In Vitro Prostate Cancer Metastasis Testbed

**DOI:** 10.1002/jbm4.10256

**Published:** 2019-12-30

**Authors:** MD Shahjahan Molla, Dinesh R Katti, Jairam Iswara, Renugopalkrishnan Venkatesan, Ramasamy Paulmurugan, Kalpana S Katti

**Affiliations:** ^1^ Center for Engineered Cancer Testbeds North Dakota State University Fargo ND USA; ^2^ Department of Civil and Environmental Engineering North Dakota State University Fargo ND USA; ^3^ Department of Urology, Saint Elizabeth's Medical Center Tufts University Boston MA USA; ^4^ Department of Chemistry and Chemical Biology Northeastern University Boston MA USA; ^5^ Department of Radiology Cellular Pathway Imaging Laboratory (CPIL), Stanford University School of Medicine Palo Alto CA USA; ^6^ Center for Life Sciences Boston Children's Hospital, Harvard Medical School, Boston Massachusetts USA; ^7^ Scintillon Institute San Diego USA

**Keywords:** PROSTATE CANCER; COLLAGEN, BONE MATRIXMATRIX MINERALIZATION, BONE MATRIXTUMOR‐INDUCED BONE DISEASE, CANCER, OSTEOBLASTS, BONE CELLS

## Abstract

In this study, two types of prostate cancer cell lines, highly metastatic PC‐3 and low metastatic MDA PCa 2b (PCa) were cultured on bone mimetic scaffolds to recapitulate metastasis to bone. A unique in vitro 3D tumor model that uses a sequential culture (SC) of human mesenchymal stem cells followed by seeding with cancer cells after bone formation was initiated to study the phenotype‐specific interaction between prostate cancer cells and bone microenvironment. The PCa cells were observed to be less prolific and less metastatic, and to form multicellular tumoroids in the bone microenvironment, whereas PC‐3 cells were more prolific and were highly metastatic, and did not form multicellular tumoroids in the bone microenvironment. The metastatic process exhibited by these two prostate cancer cell lines showed a significant and different effect on bone mineralization and extracellular matrix formation. Excessive bone formation in the presence of PC‐3 and significant osteolysis in the presence of PCa were observed, which was also indicated by osteocalcin and MMP‐9 expression as measured by ELISA and qRT‐PCR. The field emission scanning electron microscopy images revealed that the structure of mineralized collagen in the presence of PC‐3 is different than the one observed in healthy bone. All experimental results indicated that both osteolytic and osteoblastic bone lesions can be recapitulated in our tumor testbed model and that different cancer phenotypes have a very different influence on bone at metastasis. The 3D in vitro model presented in this study provides an improved, reproducible, and controllable system that is a useful tool to elucidate osteotropism of prostate cancer cells. © 2019 The Authors. *JBMR Plus* published by Wiley Periodicals, Inc. on behalf of American Society for Bone and Mineral Research.

## Introduction

Prostate cancer has a strong propensity to metastasize to bone with up to 90% of advanced‐stage prostate cancer patients having skeletal metastases.^1^ Prostate cancer is no longer curable once prostate cancer cells metastasize to bone. Multiple bone‐related complications, including bone pain, weak bones, hypercalcemia, spinal cord compression, pathological fracture, and stiffness or pain in the hip, thighs, or back, are caused by skeletal metastasis of prostate cancer. Fracture risks are greatly enhanced in men with prostate cancer.^2^ Currently, all available treatments for patients having metastasized prostate cancer are often only palliative with an aim to improve the quality of the patient's life. The 5‐year survival rate of the patients with metastasized prostate cancer is only 29%.^3^

The specific mechanisms that influence prostate cancer cells for bone metastasis are not clearly understood, although recent studies indicate that osteoblast‐rich regions are where prostate cancer cells colonize in the early stages of metastasis.^4^ After undergoing epithelial to mesenchymal transition (EMT), cancer cells escape from the primary tumor site and enter the blood circulation; they favor aggregation at the preferred remote site where they undergo mesenchymal to epithelial transition (MET) for colonization. The vein that rises from the prostate drains into the iliac vein, which then connects to the vertebral vein. The vertebral vein goes through the entire spinal column; this fact is sometimes attributed as one of the reasons behind extravasation of prostate cancer cells into the bone microenvironment,^5^ in addition to the unique chemical microenvironment of bone. Further, the hormone‐dependent nature of prostate cancer was first described in the work of Huggins and Hodges.^6^ There is evidence in the literature that suggests a link between estrogen receptor beta (ERβ) and prostate cancer metastasis. Recent studies also indicate that estrogen receptor alpha expression in prostate cancer cells is linked to osteoblastic lesion formation and lung metastasis.^7^ Clinical studies also found that ERβ is expressed in prostate cancer metastases.^8^

Of all metastases from prostate cancer, 80% spread to the bone, particularly to the axial skeleton including the spine, ribs, and pelvic bones. Although the cells spread both hematogenously and lymphatically, hematogenous spread to the bones can lead to severe pain, weakness leading to fractures, and spinal cord compression that is a clinical emergency. Current treatments include androgen deprivation, hormonal therapies such as abiraterone and enzalutamide, radionuclides, and medications to reduce skeletal events, including bisphosphonates such as zoledronate and pamidronate, and monoclonal antibodies such as denosumab. Bone metastasis is the primary cause of morbidity and mortality in prostate cancer patients. Nevertheless, the mechanisms of bone metastasis in prostate cancer are not entirely understood. To prevent bone metastasis, it is important to target not only the bone metastatic features in the tumor cells, but to block the tumor microenvironment that is nurturing tumor cells for bone metastasis as well. To advance current therapies, the effects of metastasized cancer cells on the bone microenvironment have to be better understood, and reliable in vitro models that can mimic the occurring biophysical processes in the bone are needed.

Metastasized prostate cancer cells primarily target cancellous bones. The highly vascular structure of cancellous bone provides easy access to oxygen and nutrients for metastasized prostate cancer cells, which creates a cordial environment for colonization. Bone‐forming osteoblasts and bone‐resorbing osteoclasts that reside in the bone microenvironment are responsible for creating the dynamic nature of bone. In normal healthy bone, constant remodeling of old bone by osteoclasts follows mineralization and new bone formation by osteoblasts. In the event of prostate cancer, when prostate cancer cells metastasize to bone, they can cause either excessive bone degradation in the osteoclastic lesion or excessive bone regeneration in the osteoblastic lesion. An extensive review of the bone microenvironment and its role in the migration of prostate cancer cells to bone is covered by Stewart and colleagues^9^ with some insight into next‐generation therapies for targeting the bone environment to avoid metastasis. One osteoblast‐related transcription factor abundantly studied is Runt‐related transcription factor 2 (RUNX2). The RUNX2 is a protein encoded by the RUNX2 gene in humans. RUNX2 is considered a key transcription factor associated with osteoblast differentiation and a master regulator of osteoblastic differentiation of mesenchymal stem cells (MSCs). It is the most upstream transcription factor required for differentiation to osteogenic lineage. It positively influences the early stages of osteogenic differentiation, but in the later phases the RUNX2 expression is downregulated.^10^, ^11^, ^12^, ^13^ RUNX2 is known to be aberrantly expressed in metastatic prostate cancer cells,^14^ and is also known for promoting osteolytic lesions in breast cancer at metastasis.^15^

The inability of 2D models to recapitulate entirely the complex nature of the bone–cancer microenvironment, as well as the failure of animal models to reproduce some vital characteristics specific to humans, compels the development of 3D in vitro models for systematically studying the interactions between prostate cancer cells and the bone microenvironment. In fact, several 3D in vitro models have been reported for investigating the interaction between the bone microenvironment and metastasized prostate cancer cells.^16^, ^17^, ^18^ Recent studies have investigated the interactions between human osteoblasts and prostate cancer cells in 3D tissue‐engineered bone.^19^, ^20^ Researchers have also included the design of collagen‐based scaffolds to simulate prostate cancer bone metastases.^21^ The role of α‐6 and β‐1 integrin subunits in mediating tumor–bone stromal interactions has been studied in matrigels.^22^ Species‐specific homing mechanisms of human prostate cancer metastasis in tissue‐engineered bone have been evaluated previously.^23^ Various reports in the literature on 3D co‐culture models to study prostate cancer growth, progression, and metastasis to bone,^24^ and the impact of BMP‐2 on metastasis to bone^25^ are available. Three‐dimensional in vitro models can be tailored to mimic different stages of cancer progression. Our group has developed a 3D in vitro model using tissue‐engineered scaffolds that simulate tumoroid growth and MET of prostate cancer cells during bone metastasis,^26^, ^27^ and also a testbed for developing the MET stage in breast cancer.^28^

A variety of biomaterial‐based 3D structures has been used to recapitulate bone matrix for prostate cancer metastasis, including chitosan‐alginate scaffold,^29^ alginate hydrogel,^17^ hyaluronan‐based hydrogels,^30^ collagen‐based hydrogel,^31^ polyethylene glycol‐ (PEG‐) based hydrogels,^20^ and silk fibroin scaffolds.^25^ In this study, we have used MSCs seeded polycaprolactone (PCL)/hydroxyapatite‐nanoclay‐ (HAPclay‐) based 3D scaffolds to recapitulate the bone microenvironment for prostate cancer metastasis. Prior studies from our group have led to the design of bone mimetic scaffolds using nanoclays.^32^, ^33^, ^34^, ^35^ In previous studies, we have shown that when MSCs are seeded in these scaffolds, they differentiate into bone cells without the use of osteogenic supplements.^32^ We have also reported that when prostate cancer cells are sequentially cultured with MSCs in these scaffolds, they undergo MET to form multicellular tumoroids that mimic the early colonization stage of prostate cancer bone metastasis.^36^, ^37^, ^38^ Genetic changes have been shown to be influencing both collagen and mineral in bone diseases such as osteogenesis imperfecta.^39^, ^40^, ^41^ The excessive and variant genetic changes during prostate cancer bone metastasis are suggestive of changes to the bone at metastasis. Hence, in this study, we investigated the effect of metastasized prostate cancer cells on bone regeneration, degradation, mineralization, and collagen synthesis using two different prostate cancer cell lines: PC‐3 and MDA PCa 2b. PC‐3 is a highly metastatic cell line, whereas MDA PCa 2b is less metastatic in nature.^42^ One of the most crucial components of an in vitro cancer model is the choice of cell line. There exists myriad of prostate cancer cell lines across a variety of malignancies that have been developed for preclinical studies. These cell lines vary in many of their properties, including their origin, phenotype, invasiveness, malignancy, proliferative rate, genetic background, etc. Here we utilized the PC‐3 and MDA PCa 2b cell lines to explore the differences in cell lines on various outcomes. MDA PCa 2b cells are androgen sensitive, express prostate‐specific antigen (PSA), and are noninvasive in nature. PC‐3 cells are invasive, castration‐resistant, and do not express androgen receptors and PSA. Both of the cell lines are derived from bone metastasis. Procedures such as ELISA, qRT‐PCR, immunocytochemical analysis, SEM imaging, field emission scanning electron microscopy (FESEM) imaging, and cell‐based assays were performed to investigate the effect of metastasized prostate cancer cells on the bone microenvironment. The results indicated that both osteolytic and osteoblastic bone lesions can be recapitulated in our tumor bed model.

## Materials and Methods

### Preparation of bone‐mimicking 3D porous scaffolds

In our previous studies, we have described the detailed scaffold preparation method.^32^, ^35^, ^43^, ^44^ In brief, HAP was initially mineralized inside Na‐MMT nanoclay, mimicking a biomineralization process using Na_2_HPO_4_ and CaCl_2_ by a precipitation method. We used 5‐aminovaleric acid to modify Na‐MMT clay. A freeze‐drying method was used to prepare porous scaffolds from α‐PCL and HAPclay.

### Cell lines and culture media

The human MSC line (PT‐2501) was purchased from Lonza (Walkersville, MD, USA) and maintained in MSCGM Bulletkit medium (PT‐3001; Lonza). The human prostate cancer cell line MDA PCa 2b (ATCC CRL‐2422) was purchased from American Type Culture Collection (ATCC, Manassas, VA, USA) and maintained in a medium consisting of 80% BRFF‐HPC1 (AthenaES 0403; Athena Enzyme Systems, Baltimore, MD, USA) and 20% FBS (30–2020; ATCC). The human prostate cancer cell line PC‐3 (CRL‐1435; ATCC ) was purchased from ATCC and maintained in a medium consisting of 90% HyQ Dulbecco's Modified Eagle medium DMEM‐12 (1:1) from Hyclone Laboratories (Logan, UT, USA) and 10% FBS from ATCC. All the cells were maintained at 37°C and 5% CO_2_ in a completely humidified incubator.

### Sequential cell seeding, 3D tissue culture, and tumoroid formation by sequential culture

**Figure 1:**
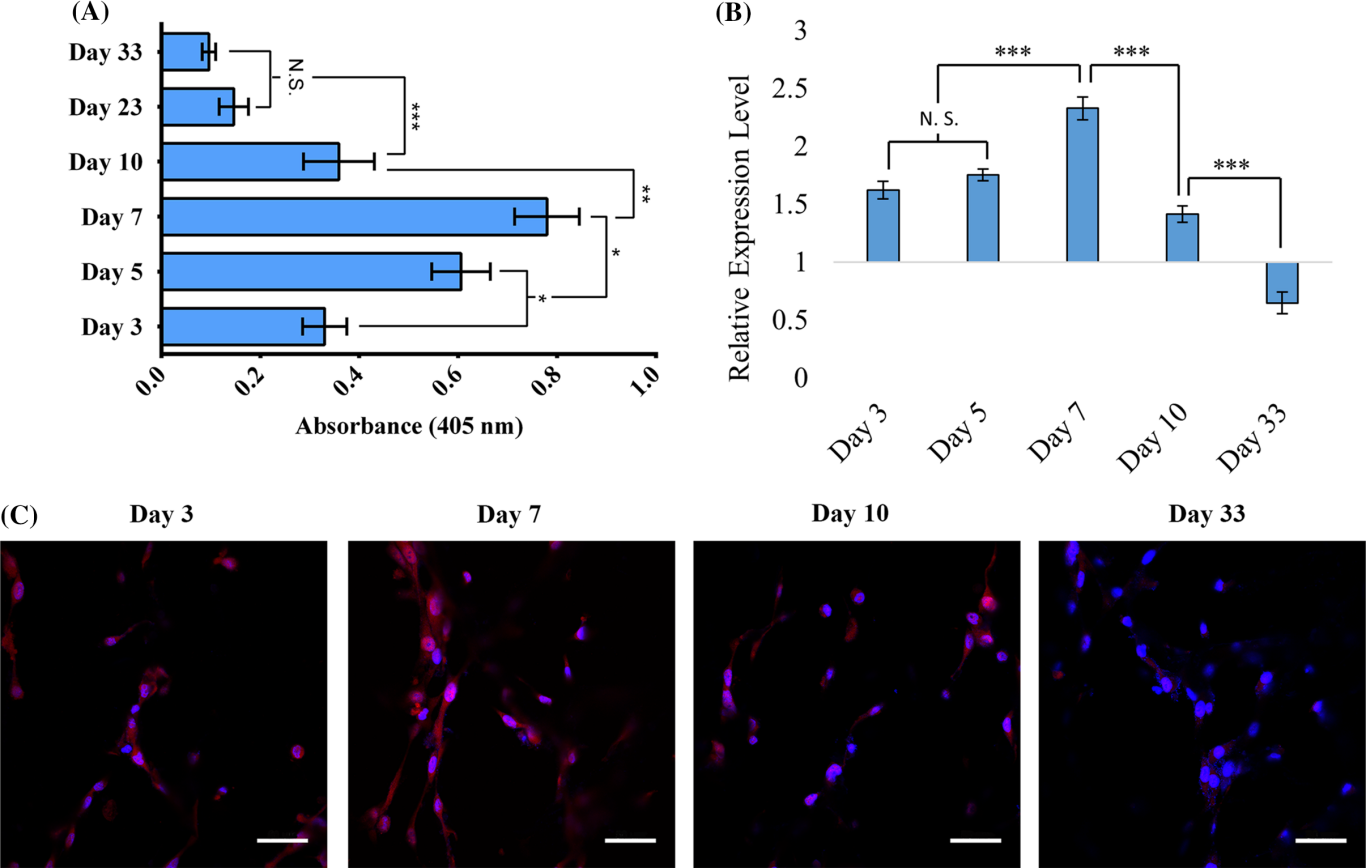
Osteogenic differentiation of mesenchymal stem cells (MSCs). (*A*) Comparative results from alkaline phosphatase assay for sequential culture (SC) and MSCs. Results are shown as mean ± SD. Statistical significance is shown by *p < 0.05; *n* = 3. (*B*) Relative gene expression level of RUNX2, which is normalized to GAPDH and where undifferentiated cultured on 2D MSCs at day 2 served as control. (*C*) Immunocytochemical analysis of Runx2 (red) and nuclei (blue) stained in MSCs cultured in 3D scaffolds.

Cylindrical disks of porous scaffolds (thickness 3 mm, diameter 12 mm) were sterilized and maintained in culture medium overnight. Human MSCs were seeded on scaffolds and cultured for 23 days (5.0 × 10^5^ cells/scaffold). Human prostate cancer cell lines MDA PCa 2b and PC‐3 cells were seeded on MSC‐seeded scaffolds after 23 days (5.0 × 10^5^ cells/scaffold) and maintained in a 1:1 mixture of MSCs and prostate cancer cells (CaP) media. CaP cells were cultured for 5, 10, and 15 days after seeding on MSC‐seeded scaffolds. In our previous work,^32^, ^37^, ^45^ we had observed that it takes at least 18 days for the MSCs to start to make mineralized bone. After 10 days, mature osteoblasts are observed. Further, mature osteoblasts start to form mineralized collagen and bone tissue. Our previous studies indicated that this entire process occurs over 23 days on the nanoclay scaffolds. So, after 23 days MSCs differentiate into bone cells; bone cells synthesize mineralized tissue that can provide PCa cells a bonelike microenvironment to mimic metastasis. Our goal was to seed the cancer cells after bonelike structures were initiated. The detailed method of SC for tumoroid formation has been described in our earlier studies.^28^, ^36^, ^37^, ^46^ In this study, the SC of PC‐3 with MSCs is referred to as PC‐3 SC, and the SC of MDA PCa 2b cells with MSCs as PCa SC (Fig. 1A). MSCs monoculture is addressed as “bone cells.”

### WST‐1 assay

**Figure 2:**
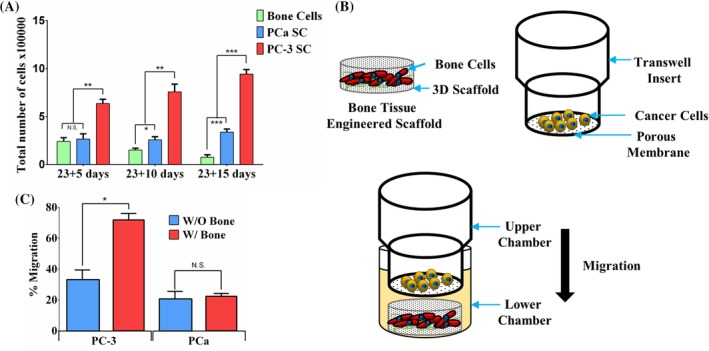
Growth and migration of prostate cancer cells. (*A*) Comparative results from WST‐1 cell viability assay for bone cells (differentiated from mesenchymal stem cells [MSCs]), PCa SC (sequential culture of MDA PCa 2b cells with MSCs), and PC‐3 SC (sequential culture of PC‐3 cells with MSCs). Results are shown as mean ± SD. Statistical significance is shown by ***p < 0.001, **p < 0.005, *p < 0.05; *n* = 3. (*B*) Schematic representation of migration assay setup. The cells were allowed to migrate towards the lower chamber (control) or bone tissue‐engineered construct (MSCs cultured in polycaprolactone/in situ hydroxyapatite‐nanoclay scaffolds for 23 days) in the lower chamber. (*C*) Percentage migration of PC‐3 cells with or without bone‐mimicking scaffolds. Results are shown as mean ± SD. Statistical significance is shown by *p < 0.05; *n* = 3.

Tetrazolium salts‐based reagent WST‐1 was purchased from Roche (Indianapolis, IN, USA). WST‐1 cell viability assay was performed for the bone cell, PC‐3 SC, and PCa SC samples on day 23 + 5, 23 + 10, and 23 + 15 (Fig. 2A). The colorimetric assay was performed following the manufacturer's protocol. Briefly, cell‐seeded samples harvested from the growth medium, washed with PBS, and placed in the reagent solution (10% in DMEM‐12), followed by incubation at 37°C for 4 hours in a humidified incubator. Absorbance was measured at 450 nm using a microplate spectrophotometer (Bio‐Rad Laboratories, Hercules, CA, USA). The total number of cells was counted using predetermined standard curves.

### Migration assay

A predetermined number of PC‐3 and MDA PCa 2b prostate cancer cells were seeded on Transwell inserts (Corning, Inc., Corning, NY, USA) of 8.0‐μm pore size in serum‐containing media. The cells were allowed to migrate towards the serum‐containing media in the lower chamber (control) or bone tissue‐engineered construct (MSCs cultured in PCL/in situ HAPclay scaffolds for 23 days) in the lower chamber as shown in Fig. 2 *B*. The total number of cells on the upper surface of the porous membrane was counted before and after migration to calculate the percentage of migration.

### Immunostaining and confocal microscopy

Immunocytochemical analysis was performed of in situ samples without harvesting cells from scaffolds. MSCs monocultures, SCs of MDA PCa 2b and PC‐3 samples were immunostained for α‐collagen. MSCs were also immunostained for RUNX2 expression. To observe the tumoroid formation with MDA PCa 2b and PC‐3 cells, SC samples were also stained for F‐actin. Samples were fixed with 4% paraformaldehyde for 30 min, and then washed three times with ice‐cold 1X PBS (Each wash for 5 min). For permeabilization, samples were treated with 0.5% Triton X‐100 in PBS for 5 min. Then 1% fish skin gelatin was used for blocking, followed by incubation with primary antibodies (ab34710 and ab76956; Abcam, Cambridge, MA, USA) at 4°C (1:150 dilution with PBS). Then, samples were washed with fresh PBS three times (Each wash for 5 min), then incubated with corresponding secondary antibodies for an hour at room temperature (Goat Anti‐Rabbit IgG H&L Alexa Fluor 488, and Goat Anti‐Rabbit IgG H&L Alexa Fluor 647; Thermo Fisher Scientific, Waltham, MA, USA). Primary and secondary antibodies were purchased from Abcam. For F‐actin staining, cell‐seeded scaffold samples were incubated with rhodamine phalloidin (R415; Thermo Fisher Scientific) for 30 min at 37°C. For nucleus staining, samples were incubated with 4′,6‐diamidino‐2‐phenylindole (DAPI) for 5 min. Z‐stacks of the scaffold samples were obtained using a Zeiss AxioObserver Z1 microscope equipped with LSM700 laser‐scanning module (Zeiss, Thornwood, NY, USA). Rendered 3D images were analyzed using Imaris software (Bitplane, South Windsor, CT, USA).

### Scanning electron microscopy and field emission scanning electron microscopy

SEM was performed to observe cellular morphology, mineralized extracellular matrix (ECM), and collagen formation using microscopes JEOL JSM‐ 6490LV for SEM and JEOL JSM‐7600F for FESEM (JEOL USA, Peabody, MA, USA). Initially, the samples were fixed with 2.5% glutaraldehyde, followed by an ethanol series treatment (10%, 30%, 50%, 70%, and 100% v/v) for dehydration. Hexamethyldisilazane was also used as a drying agent. Samples were mounted on cylindrical aluminum mounts with colloidal silver paste (Structure Probe, Inc., West Chester, PA, USA), then coated with gold (for SEM) and carbon (for FESEM). An accelerating voltage of 15 kV was used for SEM and 2.0 kV for FESEM.

### RNA extraction and qRT‐PCR analysis

Total RNAs were extracted from scaffold samples using TRI reagent (Sigma‐Aldrich, St. Louis, MO, USA) and purified using Direct‐zol RNA MiniPrep kit (Zymo Research, Irvine, CA, USA). Total RNA was quantified using Nanodrop ND 2000 (Nanodrop Products, Wilmington, DE, USA) spectrophotometer. cDNA was reverse transcribed from RNA using random primers and M‐MLV reverse transcriptase (Promega, Madison, WI, USA). SYBR Green Master MIX (Thermo Fisher Scientific) was used for template amplification with a primer for each of the transcripts by following the manufacturer's protocol in a 7500 Fast Real‐Time PCR system (Applied Biosystems, Foster City, CA, USA). The nucleotide sequences of a list of primers used in this experiment is listed in Table 1. GAPDH served as a housekeeping gene, and target gene expressions were calculated as 2^−ΔΔCt^.

**Table 1:** The Primer Sequence Used in the qRT‐PCR

Gene	Forward primer	Reverse primer
GAPDH	5′‐CATCTTCTTTTGCGTCGCCA‐3′	5′‐TTAAAAGCAGCCCTGGTGACC‐3′
COL1A1	5′‐CATCGGTGGTACTAAC–3′	5′‐CTGGATCATATTGCACA–3′
OCN	5′‐GAGCTGCCCTGCACTGGGTG‐3′	5′‐TGGCCCCAGACCTCTTCCCG–3′
MMP‐9	5′‐GGGACGCAGACATCGTCATC–3′	5′‐TCGTCATCGTCGAAATGGGC–3′
RUNX‐2	5‐CCGCCTCAGTGATTTAGGGC‐3′	5′‐GGGTCTGTAATCTGACTCTGTCC‐3′

### Alizarin Red S assay

Samples were fixed with 2.5% glutaraldehyde overnight, followed by washing with PBS three times (5 min each wash). Further, samples were stained with Alizarin Red S dye (2 g/100 mL deionized water, pH = 4.10 to 4.15). Next, samples were washed with cell culture grade water to remove excess dye. Z‐stacks of the samples were obtained using a Zeiss AxioObserver Z1 microscope equipped with an LSM700 laser‐scanning module. Rendered 3D images were analyzed using Imaris software (Bitplane, South Windsor, CT, USA).

### ELISA

The amounts of secreted MMP‐9 and osteocalcin protein were calculated using ELISA. After centrifugation at 2000 rpm, the supernatants from conditioned media were collected at day 23 + 5 and day 23 + 10 from the cell‐seeded scaffolds and used for ELISA. The level of MMP‐9 protein in the supernatants was calculated using Human MMP‐9 PicoKine ELISA kit (EK0465; Boster Bio, Pleasanton, CA, USA) following the manufacturer's protocol. The level of osteocalcin (OCN) protein in the supernatants was measured using Osteocalcin Human ELISA kit (KAQ1381; Thermo Fisher Scientific) following the manufacturer's protocol.

### Alkaline phosphatase assay

Alkaline phosphatase (ALP) assay was performed for the monoculture of MSCs seeded in the scaffolds. At day 3, 5, 7, 10, and 33 cell lysates were collected. Initially, samples were washed with PBS, and then each sample was placed in a 24‐well plate under Triton X‐100, which was purchased from Sigma‐Aldrich (X‐100). The samples were then treated with two cycles of freezing–thawing (−70°C to 37°C) to obtain cell lysates. Further, an equal volume of (250 μL) p‐nitrophenyl phosphate (N7653; Sigma‐Aldrich) was added to the cell lysate in a different 24‐well plate, then incubated for an hour at room temperature. Seventy microliters of 3 N NaOH were added to each scaffold, to deprotonate the p‐nitrophenol, then the samples were shaken for 60 s. Microplate spectrophotometer (Bio‐Rad) was used to obtain the absorbance at the wavelength of 405 nm.

### Statistical analysis

Statistical analysis was performed using Student's *t* test to compare two conditions. Differences were considered significant at *p < 0.05, **p < 0.005, and ***p < 0.001. Triplicate samples were used for performing all the experiments. Mean ± SDs were used to express quantitative data.

## Results

### Mesenchymal stem cells differentiated into bone cells

To evaluate if the MSCs differentiated into the osteoblastic lineage, we performed an ALP osteogenic differentiation assay. We also analyzed the expression of RUNX2 using qRT‐PCR assay and immunocytochemical analysis. The MSCs seeded in PCL/HAPclay scaffolds, and cell lysates were extracted at day 3, 5, 7, 10, 23, and 33 for ALP assay. A cellular membrane‐bound enzyme, ALP is a key marker for osteoblast. ALP assay is used to evaluate the differentiation of MSCs into the osteoblastic lineage. Figure 1 *A* shows a gradual increase in osteoblastic activity at the initial stage of cell seeding (from day 3 to day 7). Further, a decrease in ALP activity was observed from day 10. It has been reported that the mineralization of ECM is associated with a decreased level of ALP activity.^47^ A decrease in ALP activity of MSCs during osteogenic differentiation after day 8 has been previously reported in the literature.^48^

RUNX2 expression in MSCs cultured in 3D scaffolds was evaluated and compared with MSCs cultured on a 2D Petri dish; the result is presented in Fig. 1 *B*. To evaluate the expression of RUNX2, total RNAs were extracted from MSC‐seeded scaffolds at day 3, 5, 7, 10, and 33, and qRT‐PCR was performed. Similar to ALP activities, RUNX2 expression gradually increased from day 3 and reached peak at day 7 (Fig. 1 *B*). At day 33, the RUNX2 expression was significantly downregulated compared with control. We also evaluated the expression of RUNX2 in the MSC‐seeded scaffolds with immunocytochemical analysis at day 3, 7, 10, and 33 of the initial seeding of MSCs. The results are shown in Fig. 1 *C*. In Fig. 1 *C*, the blue color represents nuclei (DAPI) and the red color represents RUNX2 (Alexa Fluor/Thermo Fisher Scientific) of MSCs. We observed that RUNX2 staining increases from day 3 to day 7, followed by a decrease from day 7 to day 10, with very insignificant staining observed at day 33. The RUNX2 staining exhibits highest value at day 7 in MSCs cultured on PCL/HAPclay scaffolds, which is suggestive of osteoblastic differentiation. The immunocytochemical analysis is in agreement with our observation from the gene expression analysis of RUNX2, which is known to be a master regulator of osteoblastic differentiation of MSCs.^10^, ^11^, ^12^, ^13^

### Growth and in vitro migration of prostate cancer cells toward engineered‐bone tissue construct

The growth of prostate cancer cells as a function of time in the SC with bone cells was evaluated using the WST‐1 cell viability assay; the results are shown in Fig. 2 *A*. A cell viability assay was performed on day 23 + 5, day 23 + 10, and day 23 + 15. The cell population in the SCs for both of the cell lines increased over time from day 23 + 5 to day 23 + 15. Cell population in PC‐3 SC was significantly higher as opposed to PCa SC. This may result from the fact that the doubling time for MDA PCa 2b cells (42 to 73 hours) is higher than the doubling time of PC‐3 cells (approximately 33 hours).^49^ There is no significant difference between the total population of bone cells and PCa SC on day 23 + 5, which indicates that PCa cells initially may not have a significant effect on the growth of bone cells that changes over time. The initial effect of PC‐3 on the growth of bone cells cannot be determined from these data as the overall population of PC‐3 SC is more than twofold higher compared with bone cells. It is possible that PC‐3 cells have a positive effect on the growth of bone cells. The overall population of the SCs is significantly higher for both cell lines on both 23 + 10 days and 23 + 15 days, as compared with bone cells. This indicates that SC of prostate cancer cells with bone cells could be advantageous for the growth of both cancer and bone cells. The population of bone cells differentiated from MSCs decreased over the time from day 23 + 5 to day 23 + 15. Studies have indicated that MSCs can go through complex cellular events such as growth arrest during initial and terminal differentiation.^50^ Heterogeneity regarding proliferating capabilities has been observed in MSCs.^51^

The migration of prostate cancer cells into a bonelike construct is illustrated in the schematic diagram depicted in Fig. 2 *B*. Prostate cancer cells were allowed to migrate through a porous membrane to a lower chamber with or without a bone mimetic scaffold. The lower chamber without the bonelike construct served as control. The percentage of prostate cancer cells that migrated from the upper chamber into the lower chamber was calculated for each cell line, and the results are illustrated in Fig. 2 *C*. PC‐3 cells showed a higher percentage of migration with or without the bone construct as opposed to MDA PCa 2b cells. In the presence of a bone mimetic scaffold, almost 75% of PC‐3 cells migrated to the lower chamber, indicating that the cytokines and chemokines secreted from the bone cells in the bone mimetic scaffold were able to attract cancer cells toward them. For MDA PCa 2b cells, there were no significant differences in the percentage migration with or without the presence of the bone mimetic scaffold.

### Sequential culture of MDA PCa 2b forms tumoroids and PC‐3 assembles into disorganized clusters

**Figure 3:**
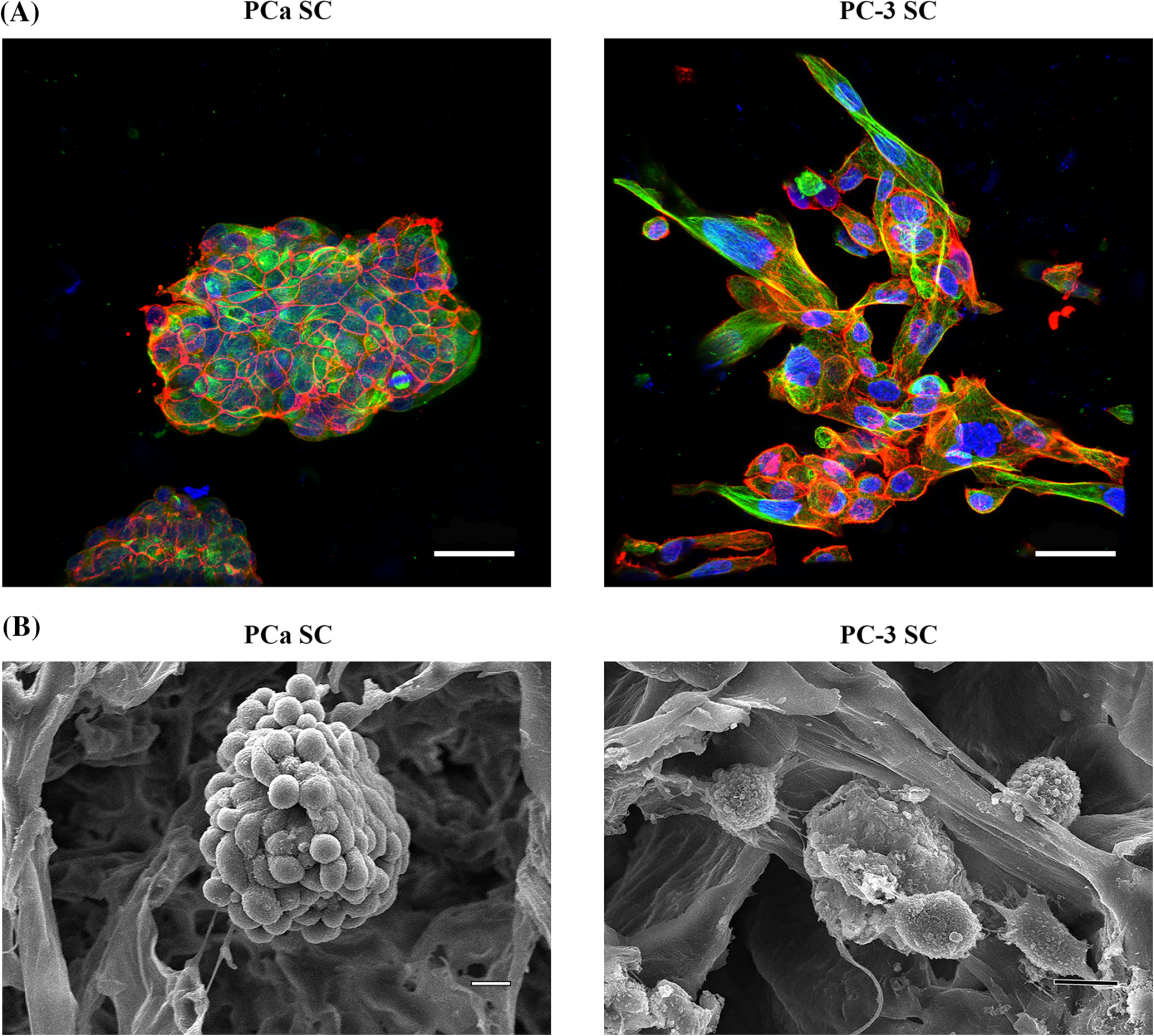
Morphological analysis of PCa SC and PC‐3 SC. (*A*) Immunostained α‐tubulin (green), F‐actin (red), and nuclei (blue) in PCa SC and PC‐3 SC. Bar = 50 μm. (*B*) SEM micrographs of PCa SC and PC‐3 SC showing tumoroid and cluster of cells, respectively, formed in the polycaprolactone/in situ hydroxyapatite‐nanoclay scaffolds. Bar = 10 μm.

To investigate the morphology of cancer cells in the tissue‐engineered bone microenvironment, we performed SEM imaging, FESEM imaging, and confocal microscopy imaging after staining the scaffolds for actin. We introduced a unique and novel cell culture technique that we term a “sequential culture” (SC).^37^ When MDA PCa 2b prostate cancer cells are sequentially cultured with human MSCs, they form multicellular organized microtissues (Fig. 3 *A*,*B*). These microtissues with hypoxic core regions are known as tumoroids. These tumoroids have tight cellular junctions and distinguishable cellular boundaries (Fig. 3 *A*,*B*). The SC of PC‐3 cells with MSCs result in aggregation of PC‐3 cells. These aggregates of cells are disorganized and exhibit a rough surface. In contrast, there is strong cell–cell adhesion in the tumoroids that are formed by MDA PCa 2b cells. When PC‐3 cells are sequentially cultured with human MSCs, they come together and a establish cell–cell adhesion; however, their cellular junctions are loose. Confocal microscopic images of immunostained MDA PCa 2b and PC‐3 cells in the SC showed a similar behavior in cancer cells. The MDA PCa 2b cells form tumoroids with tight cellular junctions, whereas PC‐3 cells form cell aggregates with loose cellular junctions.

### Mineralized bone nodule formation is enhanced in the PC‐3 metastatic site

**Figure 4:**
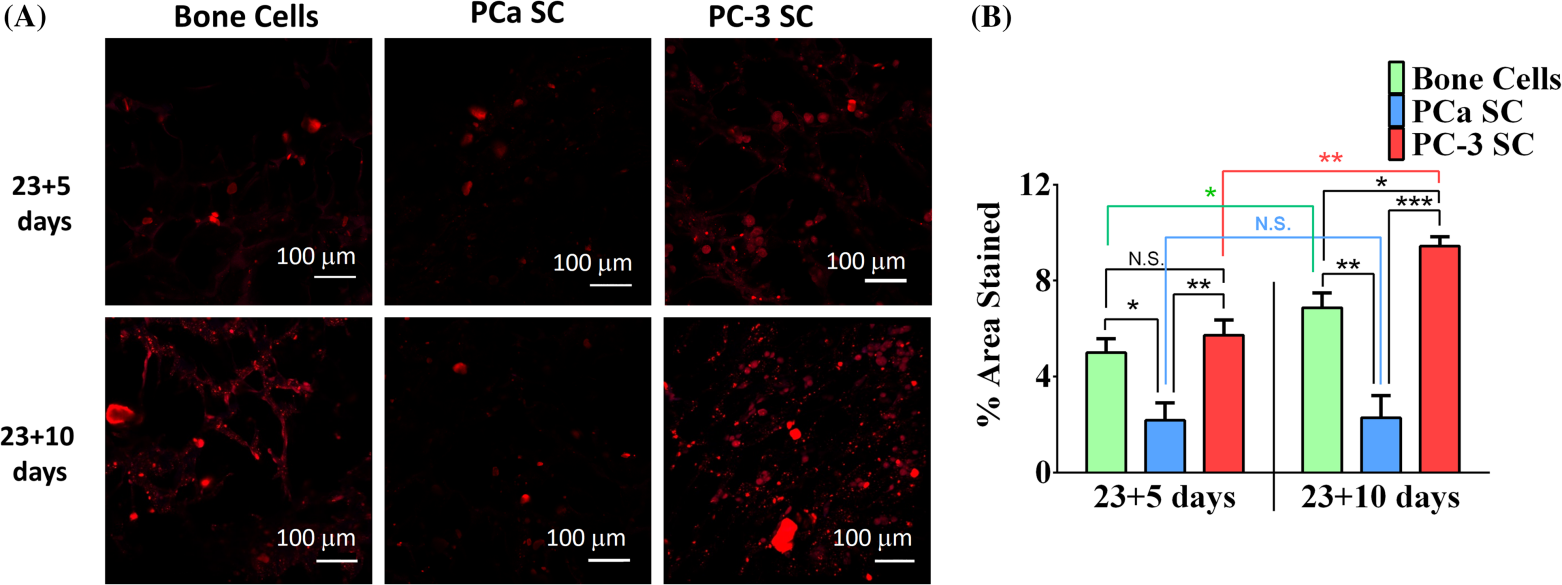
Effect on mineralization. (*A*) Alizarin Red S‐stained bone cells (differentiated from mesenchymal stem cells [MSCs]), PCa SC (sequential culture of MDA PCa 2b cells with MSCs), and PC‐3 SC (sequential culture of PC‐3 cells with MSCs) samples. Bar = 100 μm. (*B*) Calculated percentage of area stained in Alizarin Red S assay. Results are shown as mean ± SD. Statistical significance is shown by ***p < 0.001, **p < 0.005, *p < 0.05, *n* = 3.

To assess the effect of metastasized prostate cancer cells on mineralization, we performed an Alizarin Red S assay. Bone cell, PC‐3 SC, and PCa SC samples were stained; the results are shown in Fig. 4 *A*. Positive Alizarin Red S staining was observed for all the samples, indicating mineralized nodule formation. The percentage area covered by the nodules on each sample (*n* = 3) was calculated using ImageJ software (NIH, Bethesda, MD, USA; https://imagej.nih.gov/ij/); the results are presented in Fig. 4 *B*. On day 23 + 5, no significant difference in mineralization was observed between bone cells and PC‐3 SC. However, on day 23 + 10 mineralization in PC‐3 SC increased significantly compared with bone cells and PCa SC. On day 23 + 10, a branched pattern of mineralization was observed in the bone cells, which was not observed in PC‐3 SC or PCa SC. For both 23 + 5‐day and 23 + 10‐day cultures, PCa SC showed a significantly lower level of mineralization compared with bone cells and PC‐3 SC. However, there was no significant difference in mineralized nodule formation for 23 + 5 days between PC‐3 SC and bone cells. In the case of bone cells, mineralization was slightly increased from day 23 + 5 to day 23 + 10. The Alizarin Red S assay data strongly indicated that metastasized PC‐3 cells enhance the mineralized bone nodule formation. These data also suggest that calcium deposition by osteoblast cells is either decreased or stopped in the presence of MDA PCa 2b cells at the metastatic site.

### Excessive collagen synthesis at the PC‐3 metastatic site

**Figure 5:**
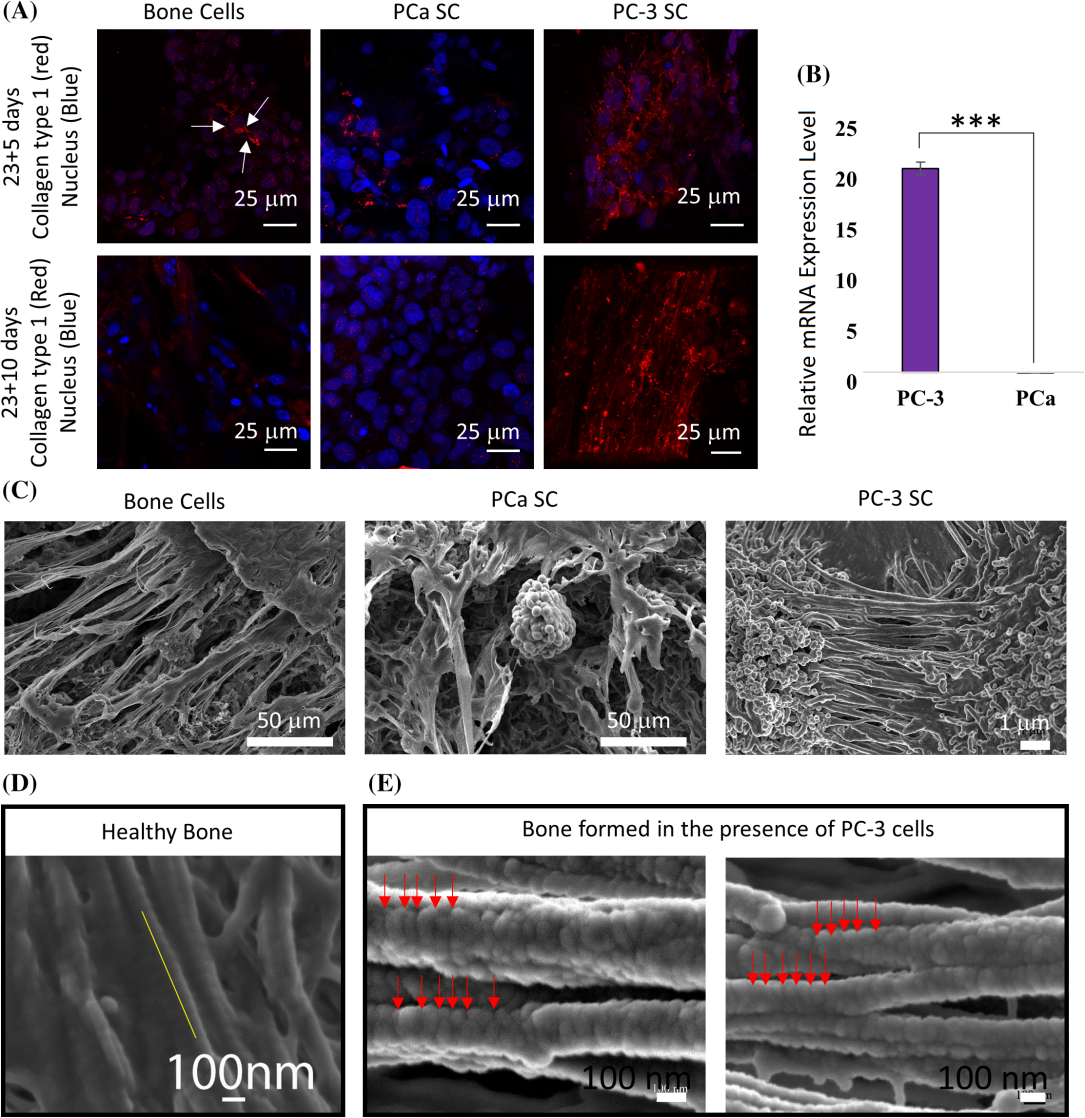
Effect on mineralized collagen formation. (*A*) Immunostained type I collagen (red), and nuclei (blue) in the bone cell (differentiated from mesenchymal stem cells [MSCs]), PCa SC (sequential culture of MDA PCa 2b cells with MSCs), and PC‐3 SC (sequential culture of PC‐3 cells with MSCs) samples. (*B*) Relative type I collagen gene expression level at day 23 + 10 in PC‐3 SC and PCa SC, where bone cells (differentiated from MSCs) served as control (relative expression = 1). Results are shown as mean ± SD. Statistical significance is shown by ***p < 0.001, *n* = 3. (*C*) SEM images of extracellular spaces of bone cell, PCa SC, and PC‐3 SC samples. (D) The 67‐nm banding pattern in the collagen fibril structure observed in healthy bone (adopted from Gu and colleagues with permission^34^). (*E*) Field emission scanning electron micrographs of mineralized type I collagen fibrils formed in PC‐3 SC.

Collagen type I is the most abundant protein in the bone ECM, accounting for up to 95% of the organic matrix. To assess the effect of metastasized prostate cancer cells on type I collagen synthesis, we performed FESEM imaging, qRT‐PCR, and immunocytochemical analysis. Figure 5 *A* shows the bone cell, PC‐3 SC, and the PCa SC samples stained with anticollagen I (red) antibody and the nuclei (blue) using DAPI. Positive staining for anticollagen I was observed for bone cells. On day 23 + 5, secreted collagen by bone cells was mostly in the monomeric form, but the initiation of collagen monomer assembly was observed (as indicated by arrows in Fig. 5 *A*). In the PC‐3 SC for the same period, anticollagen I antibody staining was significantly higher (Fig. 5 *A*), and the collagens were mostly structural, indicating faster collagen fibril synthesis in PC‐3 SC. At day 23 + 5, the PCa SC also showed positive collagen type I staining, which was noticeably less intense as opposed to the bone cells and the PC‐3 SC (Fig. 5 *A*). Collagen synthesis was increased, and monomers assembled to form a fibrillar structure in the scaffold with bone cells on day 23 + 10 (Fig. 5 *A*). These collagen fibrils are observed to be much more prolific in the PC‐3 SC samples on day 23 + 10 (Fig. 5 *A*). On day 23 + 10, the PCa SC samples showed negative type I collagen staining (Fig. 5 *A*).

Collagen gene expression was further evaluated using qRT‐PCR. Total mRNA was extracted from the bone cell, PC‐3 SC, and PCa SC samples on day 23 + 10. The resulting relative mRNA levels are plotted in Fig. 5 *B*, where the bone cell samples served as control. Collagen expression is significantly upregulated in the PC‐3 SC, but downregulated in PCa SC compared with the control. The qRT‐PCR result correlates well with our immunocytochemical observations. From the SEM images (Fig. 5 *C*), we can observe that the extracellular space of bone cell, PC‐3 SC, and PCa SC samples are very different from each other. A significant amount of ECM was observed in the SEM images of MSCs and PC‐3 SC on scaffolds, and a significantly reduced ECM was observed in scaffolds with PCa SC.

Collagen fibrils synthesized in the PC‐3 SC were analyzed using FESEM; the micrographs are shown in Fig. 5 *E*. Collagen fibrils observed in the healthy human bone samples^52^ are a few microns long (Fig. 5 *D*) and approximately 100 nm in diameter. The 67‐nm banding pattern is a unique property of mineralized collagen fibrils in healthy human bone.^53^ Our analysis of the FESEM micrographs of mineralized collagen formation in the presence of PC‐3 cells found disruption of the 67‐nm banding pattern observed in healthy bone. We observed that the distance between the bands is not uniformly 67 nm; it varies significantly with a median value of 72 nm. This is significant as it indicates disruption in bone hierarchy. Figure 5 also shows self‐assembling collagen fibrils forming collagen fibers in a parallel orientation.

### Osteocalcin is upregulated at the PC‐3 metastatic site

**Figure 6:**
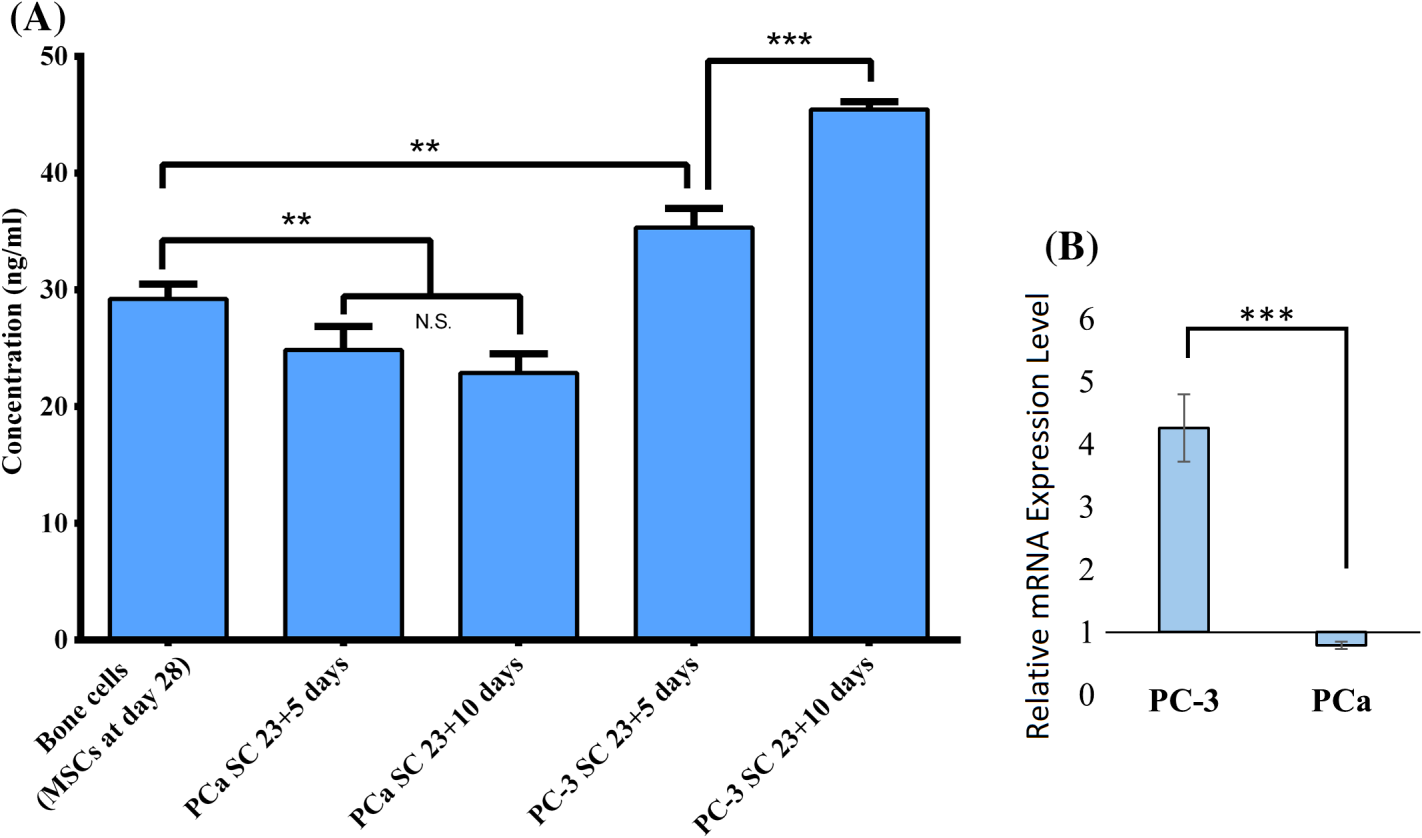
Osteocalcin (OCN) expression. (*A*) OCN protein secretion in bone cell, PC‐3 SC, and PCa SC samples measured by ELISA. Results are shown as mean ± SD. Statistical significance is shown by ***p < 0.001, **p < 0.005, *p < 0.05, *n* = 3. (*B*) Relative OCN gene expression level at day 23 + 10 in the PC‐3 SC and PCa SC samples, where bone cells (differentiated from mesenchymal stem cells) served as control (relative expression = 1). Results are shown as mean ± SD. Statistical significance is shown by ***p < 0.001, *n* = 3.

OCN is the most abundant noncollagenous bone matrix protein and is regarded as a marker of bone formation, but it seems to be involved in the process of mineralization rather than matrix production. It is primarily deposited in the ECM of bone. To investigate the effect of metastasized prostate cancer cells on the bone matrix, we analyzed protein and RNA levels of OCN using ELISA and qRT‐PCR, respectively. Total OCN protein expression was evaluated for the bone cells at day 28, and for the PC‐3 SC and the PCa SC at day 23 + 5 and 23 + 10; the results are shown in Fig. 6 *A*. OCN protein expression observed in the bone cell sample was approximately 29 ng/mL at day 28. For the same number of days, OCN protein expression was noticeably higher by approximately 5 ng/mL (approximately 34 ng/mL) in the PC‐3 SC sample. The maximum OCN protein expression was observed in the PC‐3 SC sample at day 23 + 10 (approximately 45 ng/mL). In the 5‐day period from 23 + 5 days to 23 + 10 days, OCN protein expression increased by approximately 10 ng/mL in the PC‐3 SC. OCN protein expression in PCa SC was significantly lower as opposed to bone cells and PC‐3 SC. There was no significant difference observed in OCN protein expression of the PCa SC samples at day 23 + 5 and 23 + 10. Gene expression analysis of OCN in the PC‐3 SC and PCa SC samples was performed at day 23 + 10; the results are shown in Fig. 6 *B*, where the bone cell sample served as control. OCN gene expression was significantly upregulated in PC‐3 SC as compared with control. In contrast, OCN gene expression was downregulated in PCa SC as opposed to the control. The significantly increased gene expression of OCN in PC‐3 SC correlates well with the protein expression. The increased OCN expression is likely caused by the increased expression of collagen type 1 at both the gene and ECM levels. It has been reported that increased collagen type 1 expression leads to upregulation of downstream OCN expression.^54^ It appears that OCN‐mediated extracellular mineralization of bone matrix is increased in the presence of PC‐3 cells. On the other hand, the presence of MDA PCa 2b cells has a negative effect on OCN‐mediated extracellular mineralization of bone matrix.

### Elevated levels of ECM degradation at the PCa metastatic site

**Figure 7:**
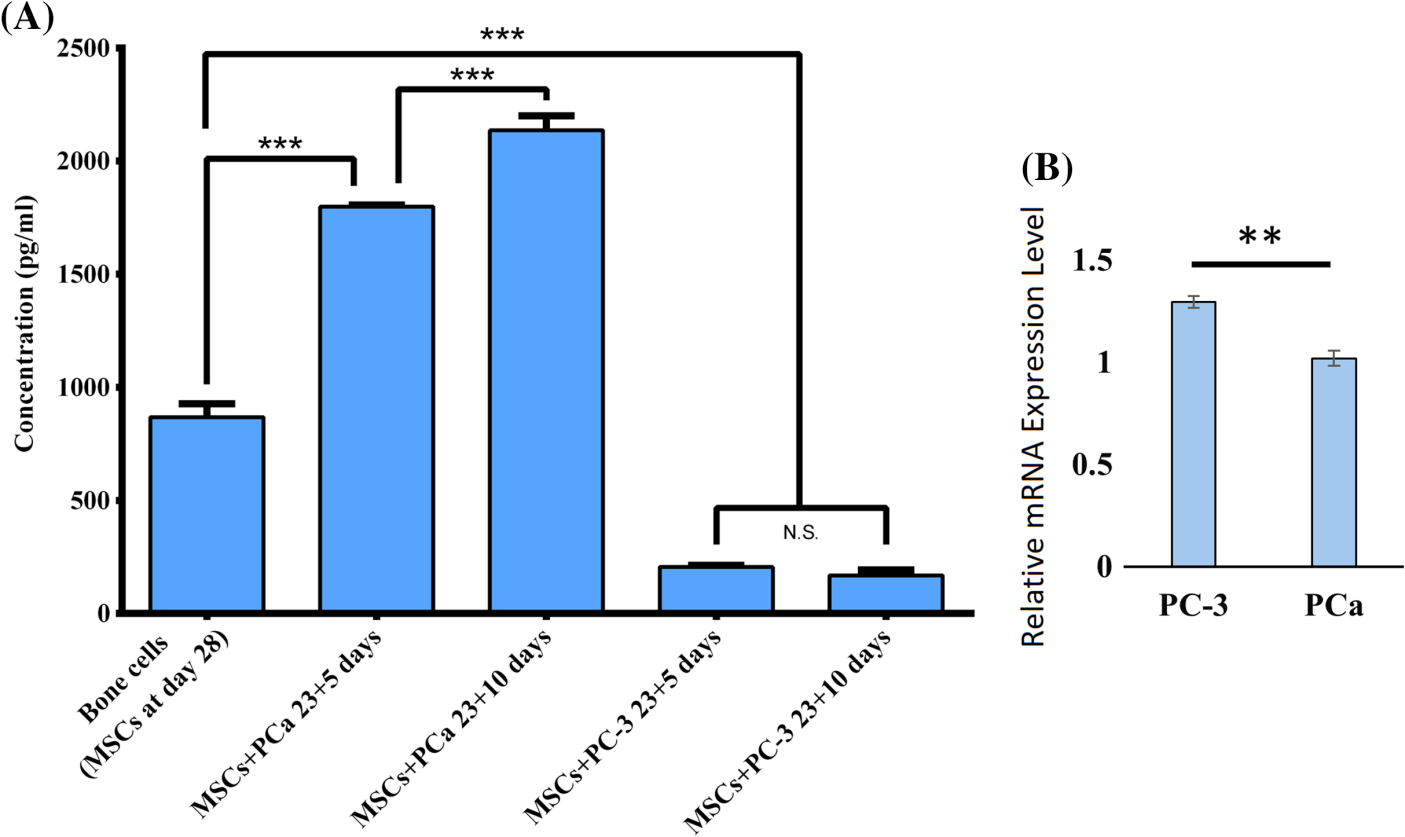
MMP‐9 expression. (*A*) MMP‐9 protein secretion in bone cell, PC‐3 SC, and PCa SC samples measured by ELISA. Results are shown as mean ± SD. Statistical significance is shown by ***p < 0.001, **p < 0.005, *p < 0.05, *n* = 3. (*B*) Relative MMP‐9 gene expression level at day 23 + 10 in PC‐3 SC and PCa SC, where bone cells (differentiated from mesenchymal stem cells) served as control (relative expression = 1). Results are shown as mean ± SD. Statistical significance is shown by **p < 0.005, *n* = 3.

One of the dominant groups of enzymes responsible for collagen and other ECM protein degradation is matrix metalloproteinases (MMPs). MMP‐9 is one of the widely investigated MMPs, which is directly associated with ECM protein degradation. MMP‐9 proteolytically processes several ECM proteins, such as collagen, fibronectin, and laminin. To investigate how metastasized prostate cancer cells play a role in ECM degradation, we evaluated the expression of MMP‐9 using ELISA and qRT‐PCR; the results are plotted in Fig. 7. The total amount of MMP‐9 excreted by the bone cells at day 28 was 868 pg/mL. Metastasized PC‐3 cells significantly inhibited the secretion of the MMP‐9 protein. MMP‐9 secretion in PC‐3 SC was significantly lower compared with bone cells and PCa SC. On day 23 + 5, MMP‐9 secreted in PC‐3 SC was approximately 206 pg/mL (Fig. 7 *A*). At the same time, MMP‐9 secretion is significantly enhanced at the PCa metastatic site compared with bone. On day 23 + 5, MMP‐9 secreted in PCa SC was approximately 1800 pg/mL as shown in Fig. 7 *A*. MMP‐9 secretion in PCa SC was further increased 5 days later at 23 + 10, which is almost 2.5‐fold higher than the secretion by bone cells. It should be noted that the same number of MSCs are initially added to each scaffold.

The MMP‐9 gene expression analysis of PC‐3 SC and PCa SC is shown in Fig. 7 *B*. Although the expression in PC‐3 SC and PCa SC was statistically significant, there was no fold‐change as opposed to the control. Prior studies do comment that a poor correlation between mRNA and its associated protein level can be observed.^50^

## Discussion

The interaction between prostate cancer and the bone microenvironment has been an important research emphasis for years because of the characteristic preference of prostate cancer cells to metastasize to bone.^9^ One of the significant barriers for investigating the osteotropic nature of prostate cancer cells has been the lack of availability of an appropriate in vitro model that mimics closely the in vivo bone microenvironment in response to bone–cancer interaction. In recent years, with the development of 3D in vitro models as tools for investigating the molecular mechanism of cancer metastasis, a significant number of novel and innovative 3D models has been reported in an attempt to recapitulate the native tumor microenvironment.^55^, ^56^, ^57^ However, none of these models used 3D SC of MSCs to generate human bone tissue on bone‐meeting scaffolds prior to seeding prostate cancer cells to recapitulate the unique remodeling of the bone environment. Our 3D SC model consists of prostate cancer cells cultured within an engineered‐bone tissue formed by differentiated MSCs. We have enabled a truly biomimetic 3D in vitro model to mimic the human bone microenvironment by using (a) a novel biomineralization route enabled with the nanoclay scaffold, (b) human MSCs instead of osteoblasts, and (c) sequential culture instead of coculture.

The ALP activity as depicted in Fig. 1 illustrates the role of osteoblast differentiation. The immunocytochemical analysis is in agreement with our observation from gene expression analysis of RUNX2, which is known to be a master regulator of osteoblastic differentiation of MSCs.^10^, ^11^, ^12^, ^13^ At the later stage of osteoblast maturation, the RUNX2 expression is inhibited. Initial upregulation of RUNX2 in MSCs cultured in 3D scaffolds evidently indicates osteogenic differentiation of MSCs at the early stage of 3D cell culture. Downregulation of RUNX2 at the later stage indicates maturation of osteoblast differentiated from MSCs, and further downregulation at day 33 (Fig. 1 *B*) indicates that mature osteoblasts turn into osteocytes.^58^ All the results from ALP assay, gene expression analysis, and immunocytochemical analysis of RUNX2 indicate the differentiation of MSCs into osteoblastic lineage without osteoblastic supplements in PCL/HAPclay scaffolds. The results also suggest that PCL/nanoclay scaffolds are osteoconductive and osteoinductive in nature.

In this study, we used two specific cell lines to evaluate the effect of metastasized prostate cancer cells on the bone microenvironment. From the 3D SC model, cell–cell and cell–matrix interactions were observed for both of the cell lines. Figure 2 shows the proliferation of prostate cancer cells in the SC and migration towards the tissue‐engineered bone, suggesting an affinity of the prostate cancer cells for the bone matrix and integration within this microenvironment. Our migration study is also in agreement with the literature because PC‐3 cells have been reported as highly metastatic in nature, whereas MDA PCa 2b cells have been identified as less metastatic.^59^

Bone cells can be induced to produce vast extracellular calcium deposits in vitro. This process is known as bone mineralization. Calcium deposits along with collagen are indications of successful in vitro bone formation. When MSCs are differentiated to osteoblasts, they can induce production of extracellular calcium deposition in vitro. Osteoblasts secrete calcium and phosphate ions into the ECM through a vesicular delivery process,^60^ which was also the context of the 2013 Nobel Prize in Medicine awarded to Rothman, Schekman, and Südhof. The vesicular delivery of mineral initiates bone mineralization. We have observed a vesicular mineralization process occurring in tissue‐engineered nanoclay scaffolds when they are seeded with human mesenchymal cells.^34^ Calcium deposits are an indication of in vitro bone formation, and can be stained bright orange‐red using Alizarin Red S. Alizarin Red S reacts with calcium cation to form a chelate. In this study, calcium deposition by bone cells was stained with bright orange‐red using Alizarin Red S (Fig. 4). These data suggest that mineralization is enhanced in the presence of PC‐3 cells, whereas MDA PCa 2b cells inhibited bone mineralization in the 3D tissue‐engineered bone model. More importantly, mineralization or osteogenic differentiation of MSCs occurred in the absence of common osteogenic agents, demonstrating the remarkable osteoinductive ability of the hydroxyapatite‐polymer‐nanoclay‐based 3D scaffolds that we have developed in our previous studies.^32^, ^61^ Osteogenic differentiation of MSCs on 3D scaffolds in the absence of osteogenic supplements has been reported in prior studies.^61^, ^62^ This is important with regard to developing materials for in vitro bone tumor model. Increased mineralization is indicative of osteoblastic osteotropism and inhibited mineralization points to osteolytic osteotropism of prostate cancer cells.

Type I collagen accounts for about 95% of the organic matrix proteins in bone. A hallmark of osteoblast differentiation is the formation of type I collagen. At day 23 + 5 collagen molecules are mainly found inside intracellular space (procollagen) in bone cells and PCa SC as shown by type I collagen staining (Fig. 5 *A*). At day 23 + 5 in PC‐3 SC some extracellular type I collagen is observed. In the extracellular space, type I collagen molecules pack together side‐by‐side, forming fibrils with a diameter of roughly 100 nm. The saturated calcium and phosphate ions secreted by osteoblasts precipitate into hydroxyapatite crystals that are cemented to this collagen fibril. In collagen fibrils from healthy bone, adjacent collagen molecules exhibit a three‐tiered hierarchy^63^ and are displaced from one another, about one‐quarter of their length, producing a characteristic pattern of bands of 67‐nm length.^64^ This characteristic 67‐nm banding pattern of mineralized collagen fibrils was observed in healthy bone in our previous study (Fig. 5 *D*).^52^ Similar collagen fibrils with a diameter of roughly 100 nm were observed in the PC‐3 SC (Fig. 5 *E*). These fibrils are several micrometers long and are packed side‐by‐side in parallel bundles. However, the banding patterns of the collagen fibrils that formed in the presence of PC‐3 cells were disrupted, and the spacing was observed to be varying and not consistently 67 nm (as shown by the red arrows in Fig. 5 *E*). This indicates the possibility of an altered collagen multiscale structure that forms in the presence of metastasized prostate cancer cells. The extracellular space of the bone cells was significantly different (Fig. 5 *C*) for the PCa SC and the PC‐3 SC samples. Collagen fibers can be observed in PC‐3 SC on 23 + 10 days and in bone cells after the same duration time. However, similar collagen fibers were not observed in PCa SC (Fig. 5 *A*). Our observation of type I collagen also correlates with the qRT‐PCR expression of type I collagen, which shows significant upregulation in PC‐3 SC as compared with PCa SC, and where bone cells served as control (Fig. 5 *B*).

OCN is an essential noncollagenous protein that binds extracellular calcium to bone matrix. It is considered a late marker for osteoblast differentiation. OCN protein expression was found in all types of samples. The data (Fig. 6 *A*) suggest that the presence of PC‐3 cells enhanced OCN expression, whereas the presence of PCa cells inhibited OCN expression. It has been reported that the increased collagen expression leads to increased downstream expression of OCN.^65^ This significantly increased protein expression of OCN in PC‐3 SC as compared with PCa SC, which correlates well with the gene expression of OCN within 10 days in SC (Fig. 6 *B*).

MMPs are key enzymes in matrix degradation. They can digest all the matrix macromolecules synergistically. MMP‐9 is one of the most extensively investigated MMPs and is directly associated with ECM protein degradation. It has been reported that MMP‐9 is able to cleave collagen I in its native form and in a manner that is characteristic of the unique collagenolytic activity of MMP collagenases.^65^ In the current study, MMP‐9 protein expression (Fig. 7 *A*) correlates well with the type I collagen expression that is shown in Fig. 5. We can conclude that the higher expression of MMP‐9‐inhibited type I collagen synthesis in PCa SC and on the other hand, enhanced type I collagen synthesis facilitated by inhibited MMP‐9 expression in PC‐3 SC. However, MMP‐9 gene expression is not consistent with the protein expression level (Fig. 7 *A*,*B*). A poor correlation between mRNA and its associated protein level has been reported before.^66^ Interpreting protein expression levels based on the gene expression level could be challenging. Changes in gene expression levels and protein levels may not correlate well, mainly because of the regulation control at different levels, ie, RNA processing, RNA stability, transcription, translation, protein stability, protein modification, and proteolytic cleavage.

All the experimental results in this study indicate osteoblastic bone formation by PC‐3 cells and osteolytic bone resorption by MDA PCa 2b, whereas PC‐3 is predominantly identified as osteolytic and MDA PCa 2b as osteoblastic in nature based on experiments at primary sites of cancer.^59^, ^67^, ^68^, ^69^ ex vivo studies have demonstrated the osteoblastic characteristic of PC‐3 cells in a 3D cancer–bone model using a co‐culture of live calvarial bones and cancer cells.^16^ A mixed (osteoblastic and osteolytic) characteristic of PC‐3 cells in an in vitro 3D bone model by using human bone tissue culture was also shown.^70^ The induction of osteoblastic activity of the PC‐3 cell by inhibiting DKK‐1 has also been reported.^71^ Bone matrix proteins, OCN and osteopontin play important roles in the growth and progression of metastatic prostate cancer.^72^ An increase in bone‐related genes, type I collagen, osteonectin, osteopontin, and ALP was observed in the co‐culture of PC‐3 with human osteoblasts.^73^ The osteolytic nature of PC‐3 cells in a humanized tissue‐engineered in vivo model where PC‐3 cells undergo EMT in the bone microenvironment has been reported.^67^ In a real bone microenvironment, prostate cancer cells undergo MET, which is the reverse process of EMT. In a previous study, we have shown that PC‐3 cells undergo MET in our tissue‐engineered bone model, indicating the more biomimetic nature of our 3D in vitro model with respect to the native human bone microenvironment.^26^ The protein expression, gene expression, and immunocytochemistry results of this study indicate osteolytic bone resorption by MDA PCa 2b cells. To the best of our knowledge, this is the first time an osteolytic bone resorption phenomenon has been observed with the MDA PCa 2b cancer cell line either in in vivo or in vitro studies. However, other studies have reported mixed osteoblastic and osteolytic activity of MDA PCa 2b cells^59^; when MDA PCa 2b were co‐cultured with osteoblasts, OPG expression was inhibited and RANKL expression was enhanced. In light of our observation in this study, we conclude that osteolytic or osteoblastic bone metastasis occurrence depends not only on the cancer type, but is also significantly influenced by the bone microenvironment of the cells.

## Conclusions

In summary, we have investigated the effect of metastasized prostate cancer cells on bone mineralization and ECM formation using an in vitro 3D tumor model. Two different prostate cancer cell lines, a highly metastatic PC‐3 and a nonmetastatic MDA PCa 2b (PCa) were used to create metastasis tumors at the bone site. Our experimental results indicate large differences in the influence of the two prostate cancer cell lines at bone metastasis. We observed that at the bone site, PCa is less prolific and less metastatic, and forms multicellular tumoroids in the bone microenvironment. The PC‐3 cell line is more prolific, highly metastatic, and does not form multicellular tumoroids in the bone microenvironment. The metastasized prostate cancer cells exhibit a significant effect on bone mineralization and ECM formation. Experiments with Alizarin Red S staining and collagen type I expression analysis indicate that excessive bone formation occurs in the presence of PC‐3, and significant osteolysis results in the presence of PCa. This observation was further confirmed by an osteocalcin and MMP‐9 expression analysis using ELISA and qRT‐PCR. FESEM images revealed that the structure of collagen, which formed in the presence of PC‐3, to be altered from that of healthy bone. All the experimental results indicate that both osteolytic and osteoblastic bone lesions resulting from the different cell lines can be recapitulated in our tumor model. Our in vitro tumor model does not entirely recapitulate the bone tumor microenvironment found in a patient with metastatic disease, but with the inclusion of human MSCs and the SC technique, it certainly provides an improved reproducible and controllable system that is a useful tool for evaluating the osteotropism of prostate cancer cells. The model may be further extended by including additional human components, such as human hematopoietic stem cells, to provide osteoclast and immune cells in the bone microenvironment. This model provides an intriguing opportunity to dissect the role of genes, proteins, and other factors in the bone microenvironment subsequent to the arrival of prostate cancer cells, with a significant contribution to the understanding of the molecular, cellular, and mechanical mechanisms.^74^ The model can be used to investigate the various aspects of prostate cancer–bone interaction at a cellular, molecular, and genetic level, as well as to test various cancer therapeutics.

## Disclosures

All authors of this article state that they have no conflicts of interests.
